# Contribution to the Pathological Anatomy of Chorea, with the Report of a Case

**Published:** 1890-09

**Authors:** 


### CONTRIBUTION TO THE PATHOLOGICAL ANATOMY OF CHOREA, WITH THE REPORT OF A CASE.

Dana (*Brain*, 1890, Spring Number, 71) reports a case of chorea in a youth of eighteen years, who had suffered from the affection for twelve years. He had also had occasional epileptic attacks for several years. He lay in bed unable to walk or help himself on account of the violence of the choreic movements. His expression was dull, and his speech slow and jerky, on account of the movements of the lips and tongue. Under the influence of treatment the choreic movements and the hysteroid or epileptic attacks diminished, but the patient died of pneumonia in less than a month after coming under observation.

The autopsy showed nothing of importance wrong, on microscopic examination. Microscopic study, however, revealed a non-adhesive leptomeningitis of the brain, with diffuse and varicose dilatations of the small arteries, especially of the deeper sub-cortical matter and capsule. There were degenerative changes in the arterial walls, and great dilatation of the perivascular spaces. The cortical cells were in most regions normal. The severest changes, vascular, interstitial, and degenerative, were in the under surface of the temporal lobes, the internal capsule, and adjacent parts of the corpus striatum (especially lenticular nucleus and optic thalamus, antero-internal part). Varicose nerve fibres were here noticed. In the pons and medulla the same condition was observed, though less marked. There was cell degeneration in some of the cranial nerve nuclei, and slight connective tissue increase in the pyramidal tracts.

In the spinal cord there was slight leptomeningitis and congestion of the cord, especially in the lateral tracts. There was also a double central canal in certain portions.

The author has made a careful study of the literature of the subject, and has examined the post mortem records in over eighty cases, but a critical analysis showed him that there are only thirty-nine of these available for study, as the others were either not instances of subacute or chronic chorea, or no satisfactory account of the lesions of the nervous system is given, or other reasons necessitated their exclusion from the tabulated arrangement which the article contains.

A review of these cases shows that in subacute chorea there is hyperæmia of the brain and parts of the cord. In the brain this is not meningeal, but subcortical and basal. The arterial walls are paralyzed, dilated, and badly nourished; exudations occur, and the lymph-spaces become distended and eroded. There are stasis, thrombosis, spots of softening, minute hemorrhages. The lymph spaces around the ganglion cells are not dilated.

In chronic cases the vascular and neuro degenerative changes are more marked. The small arteries are permanently dilated, thickened and degenerated, and the perivascular channels eroded and distended. There is also some connective tissue proliferation, and signs of degeneration in the ganglionic cells. The nerve fibres show a varicosity. Hyaline bodies are seen. The process is, in fine, first a vaso-motor paralysis and trophic disturbance affecting certain areas of the brain, and, to a less extent, of the cord. Then we have this becoming chronic, with connective tissue hyperplasia, and degenerative changes in ganglionic cells and fibres. The disease cannot be localized in any one spot. It is rather a disease of the intracranial motor tract, including its starting point in the cortex, and especially in its co-ordinating adjuncts, the lenticular nucleus and thalamus.—*American Journal of the Medical Sciences.*

